# Outcome of spinal implant-associated infections treated with or without biofilm-active antibiotics: results from a 10-year cohort study

**DOI:** 10.1007/s15010-020-01435-2

**Published:** 2020-05-05

**Authors:** Karolin Köder, Sebastian Hardt, Max S. Gellert, Judith Haupenthal, Nora Renz, Michael Putzier, Carsten Perka, Andrej Trampuz

**Affiliations:** Corporate Member of Freie Universität Berlin, Humboldt-Universität zu Berlin, and Berlin Institute of Health, Center for Musculoskeletal Surgery (CMSC), Charité - Universitätsmedizin Berlin, Charitéplatz 1, 10117 Berlin, Germany

**Keywords:** Spine, Implant, Infection, Biofilm, Outcome

## Abstract

**Purpose:**

Biofilm-active antibiotics are suggested to improve the outcome of implant-associated infections; however, their role in infections after spinal instrumentation is unclear. Therefore, we evaluated the outcome of patients with spinal implant-associated infections treated with and without biofilm-active antibiotics.

**Methods:**

The probability of infection-free survival was estimated for treatment of spinal implant-associated infections with and without biofilm-active antibiotics using the Kaplan–Meier method; Cox proportional-hazards regression model was used to identify factors associated with treatment failure.

**Results:**

Among 93 included patients, early-onset infection was diagnosed in 61 (66%) and late-onset in 32 infections (34%). Thirty patients (32%) were treated with biofilm-active antibiotic therapy and 63 (68%) without it. The infection-free survival after a median follow-up of 53.7 months (range, 8 days-9.4 years) was 67% (95% confidence interval [CI], 55–82%) after 1 year and 58% (95% CI 43–71%) after 2 years. The infection-free survival after 1 and 2 years was 94% (95% CI 85–99%) and 84% (95% CI 71–93%) for patients treated with biofilm-active antibiotics, respectively, and 57% (95% CI 39–80%) and 49% (95% CI 28–61%) for those treated without biofilm-active antibiotics, respectively (p = 0.009). Treatment with biofilm-active antibiotics (hazard ratio [HR], 0.23, 95% CI 0.07–0.77), infection with *Staphylococcus auras* (HR, 2.19, 95% CI 1.04–4.62) and polymicrobial infection (HR, 2.44, 95% CI 1.09–6.04) were significantly associated with treatment outcome. Severe pain was observed more often in patients without biofilm-active antibiotic therapy (49% vs. 18%, *p* = 0.027).

**Conclusion:**

Treatment with biofilm-active antibiotics was associated with better treatment outcome and less postoperative pain intensity.

**Electronic supplementary material:**

The online version of this article (10.1007/s15010-020-01435-2) contains supplementary material, which is available to authorized users.

## Introduction

Instrumented spinal surgeries are increasingly performed in children and adults [4, 8, 18, 20]. The incidence of infections following spinal fusion surgery ranges from 0.5% [1], 2.6 to 3.8% [7, 26] and up to 10% [17, 22, 23]. The wide range of infection rate may reflect absence of standardized definition criteria, different diagnostic approaches, various follow-up periods and heterogeneous patient populations [9]. The burden of spinal implant-associated infections is high, requiring additional surgical interventions, prolonged antimicrobial treatment, extended hospital stays and may cause long-term disability, chronic pain and potentially irreversible spine injury [6, 11, 31, 32].

The treatment goal of spinal implant-associated infections is a pain-free, mobile patient with spinal stability and eradicated infection. Most authors agree that in early-onset infections with well-fixed and functional spinal implants, the device should be debrided and retained [10, 14], despite some authors suggest to always remove the implants to achieve cure of infection [3, 7, 17]. In late-onset implant-associated infection, typically presenting in an indolent slowly progressive nature, high treatment failure rates is reported with implant retention [5, 29]. Therefore, in late-onset infections with loose implants and bony fusion, implants should be removed [1, 14–17, 32]. It remains controversial, whether only the loose parts or the complete implant should be exchanged, taking into account considerable morbidity associated with complete removal [12, 13].

In addition to the surgical therapy, it remains unclear which antibiotics should be used to achieve eradication of infection, especially the role of biofilm-active antibiotics is unknown [33]. Antimicrobial treatment is currently prescribed mainly based on personal experience, institutional tradition, local recommendations and, therefore, largely differs between individual institutions, professional disciplines and countries [28].

We evaluated the characteristics and outcome of spinal implant-associated infection, treated with and without biofilm-active antibiotics, regardless of the surgical therapy, using uniform definition criteria for infection and active long-term follow-up evaluation.

## Materials and methods

### Ethics statement

The study protocol was reviewed and approved by the institutional ethics committee (EA2/040/13) and performed in accordance with the Declaration of Helsinki. Patients provided informed consent for the inclusion in the institutional spinal infection cohort.

### Study population

The study was conducted in a tertiary healthcare center, providing advanced specialty care to about four million inhabitants. Patients diagnosed with infection after implantation of spinal hardware were prospectively included in the cohort. Spinal infections are systematically documented as part of the institutional quality-assurance program.

### Study design

Patients were included in the study and followed-up prospectively, whereas the treatment outcome was retrospectively evaluated by an interdisciplinary team consisting of orthopedic surgeons and infectious diseases specialists. The team evaluated the outcome of each episode according to predefined criteria and was blinded regarding the use of biofilm-active antibiotics. Excluded were patients with incomplete dataset or isolation of one or more difficult-to-treat infections, defined as infections caused by rifampin-resistant staphylococci, ciprofloxacin-resistant gram-negative bacilli and fungi, for which no biofilm-active antibiotics exist. During the study period, surgical techniques, instruments, implant types or diagnostic procedures remained unchanged.

### Definition of infection

For this study, definition criteria of the Centers for Diseases Control and Prevention (CDC) for vertebral disc space infection [30] were modified, as proposed by Kowalski et al. [14] and Dubée et al. [10]. Spinal implant-associated infection was defined by presence of at least one of the following criteria: (i) microbial growth from intraoperative tissue or abscess obtained during percutaneous or open biopsy, or from sonication of removed spinal implant, (ii) intraoperative purulence or secondary wound dehiscence with implant on view, (iii) radiographic evidence of inflammation on x-ray, computed tomography (CT), magnetic resonance imaging (MRI) or radiolabeled scan with gallium or technetium and either systemic signs of inflammation such as fever (> 38 °C) without other recognized cause or increasing pain at the site of spinal instrumentation.

Low-virulent microorganisms, such as coagulase-negative staphylococci, *Corynebacterium* spp. or *Cutibacterium* spp. were considered causative pathogens, if isolated in at least two independent samples or another non-microbiological criterion was present. According to the time between primary implantation and onset of infection, postoperative infections were classified as early-onset (≤ 30 days) or late-onset (> 30 days). If documented, hematogenous spinal implant-associated infections were documented separately.

### Data collection

Data were extracted from electronic medical charts into a standardized case report form. The following data were extracted: age, sex, body height and weight, coexisting medical conditions, reason for primary spinal surgery, anatomic site of spinal surgery, date of implantation and diagnosis of infection, clinical findings (fever, neck or back pain, local signs of inflammation, tenderness on percussion, sinus tract, focal neurological impairment), radiological, laboratory and microbiological findings. Coexisting medical conditions were defined as follows: diabetes mellitus, chronic renal failure (defined with estimated glomerular filtration rate < 60 ml/min present for > 3 months), active malignancy, immunosuppression (HIV infection with < 200 CD4 + lymphocytes/mm^3^ or use of > 25 mg prednisone-equivalent/day or other immunosuppressive medication in the preceding month), exposure to radiotherapy or chemotherapy, liver cirrhosis (defined by histopathological pattern).

### Definition of biofilm-active antibiotics

The initial antibiotic treatment consisted of empiric broad-spectrum intravenous treatment, followed by targeted intravenous therapy for 1–3 weeks and then oral antibiotics for a total duration of 12 weeks, except for streptococci and enterococci, where oral therapy was prolonged to 6–12 months (see below). Biofilm-active antibiotics were started after the wound discharge ceased and the surgical drains were removed. The antibiotic treatment with biofilm-active antibiotics is summarized in the “Pocket Guide to Diagnosis and Treatment of Spinal Infections” (see Supplementary Material).

In brief, for staphylococci [34], *Cutibacterium* spp. [19] and culture-negative infections biofilm-active antibiotics included rifampin in combination with intravenous flucloxacillin, vancomycin/daptomycin or fosfomycin, followed by oral quinolones (levofloxacin, ciprofloxacin or moxifloxacin), cotrimoxazole, doxycycline or fusidic acid, depending on the antimicrobial susceptibility of the pathogen. For quinolone-susceptible gram-negative bacteria, oral ciprofloxacin was used [25]. For streptococci, intravenous penicillin G or ceftriaxone was initially used and for enterococci, intravenous ampicillin or vancomycin/daptomycin was initially used, both types of pathogens were subsequently treated with oral amoxicillin (or alternative antibiotic such as doxycycline, cotrimoxazole or levofloxacin/moxifloxacin, depending on the antimicrobial susceptibility or allergy).

Against streptococci and enterococci, oral antibiotic treatment was prolonged to 6–12 months to eradicate the biofilm, as rifampin exhibits no biofilm activity on these pathogens. This prolonged treatment approach recommendation was extrapolated from the treatment of periprosthetic joint infections [2, 21, 24] and was considered as “biofilm-active” for the purpose of the present study.

In polymicrobial infections, a combination of biofilm-active antibiotics were used to treat all isolated pathogens according to their antimicrobial susceptibility.

### Outcome evaluation

Patients were scheduled for follow-up visits at 3, 6 and 12 months after surgery using a standardized case report form. Clinical signs or symptoms of infection, intercurrent surgical intervention, antimicrobial use and pain intensity were documented. For pain evaluation the Numerical Rating Scale (NRS) was used, where 0 means no pain and 10 the worst pain imaginable. Patients who did not appear at scheduled appointment were contacted by phone or information was obtained by their general practitioner. Infection-free state was defined, if all of the following criteria were present: (i) no clinical or radiological signs of inflammation (such as osteolysis or implant loosening), (ii); no subsequent surgical intervention for infection, (iii) no infection-related death and (iv) no antimicrobial suppression therapy.

### Statistical analysis

Categorical variables were compared using *χ*^2^ or Fisher’s exact tests, as appropriate. To test for the difference between two medians, the Mann–Whitney *U* test was used. The probability of infection-free survival and 95% confidence interval (95% CI) was estimated using the Kaplan–Meier survival method and groups were compared by log-rank test. The influence of individual variables on the infection-free survival was analyzed by a univariate and multivariate Cox proportional-hazards regression model for the total duration of the follow-up. The hazard ratio (HR) with 95% CI was calculated by the Akaike information criterion using forward and backward selection. A *P* value (two-sided) < 0.05 was considered significant. For the sample size calculation, the following parameters were used: power 90%, *α* = 5%, a significance level 5% (one-sided), drop-out rate 20%. The proportion of relapse-free patients within first year after surgery was estimated to be 85% with biofilm-active antibiotics and 70% for those treated without biofilm antibiotics, i.e. a non-inferiority margin of *δ* = −10%. Using these parameters, a sample size of 84 patients was estimated. For statistical analysis the program R (version 3.1.3., available from: https://www.R-project.org/.) and for graphics the software Prism (version 8.2; GraphPad, La Jolla, CA) was used. Sample size calculation was performed with the nQuery Advisor^®^ (version 7.0).

## Results

### Patient characteristics

A total of 104 patients with infected spinal implants were identified during the study period. After exclusion of eight patients with difficult-to-treat pathogens (three rifampin-resistant coagulase-negative staphylococci, three ciprofloxacin-resistant gram-negative bacilli, two *Candida albicans*) and three patients with incomplete dataset, 93 patients were analyzed.

Table 1 summarizes the demographic and primary spine surgery data of 93 included patients, stratified into 30 patients treated with biofilm-active antibiotics (32%) and 63 patients treated without biofilm-active antibiotics (68%). Baseline characteristics were similar in both groups except for the indication for primary spine surgery, where degenerative spine diseases (*p* = 0.050) was more common in the group not receiving biofilm-active antibiotics and vertebral osteomyelitis (*p* = 0.013) was more common in the group receiving biofilm-active antimicrobials. As primary spine surgery, dorsal lordosing spondylodesis was performed in 74 patients (80%); 37 patients (40%) underwent more than one previous surgery. The number of stabilized spinal segments ranged from 1 to 17 (median, 4 segments).

**Table 1:** Characteristics of 93 patients with spinal implant-associated infections

Characteristic	All patients (*n* = 93)	Patients treated with biofilm-active antibiotics (*n* = 30)	Patients treated with biofilm-nonactive antibiotics (*n* = 63)	*P* value
Age, median (range)—years	66 (11–85)	68 (11–83)	66 (18–85)	0.822
Female sex	46 (49)	13 (43)	34 (54)	0.380
Body mass index, median (range), kg/m^2^	28.5 (14.6–38.1)	28.3 (14.6–37.0)	28.6 (18.7–38.1)	0.714
Body mass index ≥ 25 kg/m^2^	56 (60)	15 (50)	43 (68)	0.523
ASA, median (range)	2 (1–4)	3 (2–4)	2 (1–4)	0.425
Comorbidities, median (range)	2 (0–6)	2 (0–6)	1 (0–5)	0.233
Coexisting medical conditions^a^				
Arterial hypertension	51 (55)	17 (57)	34 (54)	0.828
Diabetes mellitus	25 (27)	12 (40)	13 (21)	0.078
Active malignancy	21 (23)	8 (27)	13 (21)	0.598
Rheumatic or other autoimmune disease	13 (14)	4 (13)	9 (14)	1.000
Hypothyroidism	10 (11)	2 (7)	8 (13)	0.492
Chronic renal failure	9 (10)	3 (10)	6 (10)	1.000
Radiotherapy or chemotherapy	7 (8)	1 (3)	6 (10)	0.422
Immunosuppression	4 (4)	1 (3)	3 (5)	1.000
Liver cirrhosis	3 (3)	2 (7)	1 (2)	0.243
Indication for primary spinal surgery				
Degenerative spinal disease	45 (48)	10 (33)	35 (56)	0.050
Vertebral fracture	20 (22)	6 (20)	14 (22)	1.000
Spinal tumor	15 (16)	5 (17)	10 (16)	1.000
Vertebral osteomyelitis	8 (9)	6 (20)	2 (3)	0.013
Congenital deformity	5 (5)	3 (10)	2 (3)	0.324
Level of spine stabilization^b^				
Cervical	6 (6)	2 (7)	4 (6)	1.000
Thoracic	35 (38)	13 (43)	22 (35)	0.495
Lumbosacral	52 (56)	15 (50)	37 (59)	0.510
Stabilized segments, median (range)	4 (1–17)	5 (2–15)	3 (2–17)	0.106

Data are *n*. (%) of patients, unless otherwise indicated

*ASA* American Society of Anesthesiology

^a^Definitions of coexisting medical conditions are summarized in Methods

^b^Categorized by the most superior segment involved

### Infection characteristics

Table 2 summarizes the infection characteristics. Early-onset infection was diagnosed in 61 infections (66%) and late-onset in 32 infections (34%). No hematogenous infection was diagnosed. Most common clinical signs were neck or back pain (75%), local inflammatory signs at incision site (74%) and tenderness on percussion (62%). Fever, sinus tract or focal neurologic impairment were observed in < 10% of spinal implant-associated infections. Radiologic signs of inflammation on spine CT or MRI were found in 21 of 52 patients (40%), in whom the imaging was performed. The preoperative serum C-reactive protein (CRP) value was increased (> 10 mg/l) in 89% of infections and the white blood cell count (> 10 × 10^9^/l) in 36%. No differences in CRP or white blood cell counts were observed between early and late infections. The time from infection diagnosis to surgical revision ranged from 0 to 26 days (median, 7 days).

**Table 2:** Infection characteristics of 93 patients with spinal implant-associated infections

Characteristics	All patients (*n* = 93)	Patients treated with biofilm-active antibiotics (*n* = 30)	Patients treated with biofilm- nonactive antibiotics (*n* = 63)	*p* value
Time of infection onset after surgery				0.817
Early-onset (≤ 30 days)	61 (66)	19 (63)	42 (67)	
Late-onset (> 30 days)	32 (34)	11 (37)	21 (33)	
Time between implantation and infection onset, median (range)—days	20 (1–3672)	16 (5–369)	21 (1–3672)	0.238
Clinical findings Fever > 38 °C Neck or back pain Local inflammatory signs^a^ Tenderness on percussion Presence of sinus tract Focal neurological impairment^b^	7 (8) 70 (75) 75 (81) 58 (62) 6 (7) 4 (4)	4 (13) 19 (63) 24 (80) 17 (57) 1 (3) 0 (0)	3 (5) 51 (81) 51 (81) 41 (65) 5 (8) 4 (6)	0.207 0.077 1.000 0.495 0.660 0.301
Radiological findings on spine imaging (CT or MRI) Implant loosening Osteolysis or bone defect Abscess^c^	21/52 (40) 8/52 (15) 7/52 (13) 6/52 (12)	7/21 (33) 4/21 (19) 1/21 (5) 2/21 (10)	14/31 (45) 4/31 (13) 6/31 (19) 4/31 (13)	0.563 0.700 0.093 1.000
Laboratory findings before surgery Serum CRP value > 10 mg/l White blood cell count > 10 × 10^9^/l	71/80 (89) 29/80 (36)	26/28 (93) 12/28 (43)	45/52 (87) 17/52 (33)	0.483 0.466
Length of hospital stay, median (range), days	18 (3–103)	26 (4–103)	17 (3–100)	0.064

Data are *n*. (%) of patients, unless otherwise indicated. Whenever a denominator is shown, data are not available for all patients

*CRP* C-reactive protein, *CT* computed tomography, *MRI* magnet resonance imaging

^a^Wound dehiscence or discharge, redness or warmth at the incision site

^b^Including paresthesia (*n* = 2) and paresis (*n* = 2)

^c^Including paravertebral (*n* = 4) and epidural abscess (*n* = 2)

### Microbiological findings

The pathogen was identified in 72 infections (77%), including 59 (14%) monomicrobial and 13 (23%) polymicrobial infections (Table 3). Among monomicrobial infections, *S. aureus* (*n* = 32), coagulase-negative staphylococci (*n* = 16) and enterococci (*n* = 8) were the predominant pathogens. Streptococci (*n* = 3) were isolated only in polymicrobial infections. The most common pathogens in polymicrobial infections were gram-negative bacilli, including *Enterobacter* spp., *E. coli* and *P. aeruginosa*.

**Table 3:** Microbiological findings of 93 patients with spinal implant-associated infections

Pathogen	All patients (*n* = 93)	Patients treated with biofilm-active antibiotics (*n* = 30)	Patients treated with biofilm-nonactive antibiotics (*n* = 63)	*p* value
*Staphylococcus aureus*^a^	32 (34)	8 (27)	24 (38)	0.353
Coagulase-negative staphylococci^b^	16 (17)	5 (17)	11 (17)	1.000
Enterococci	8 (9)	7 (23)	1 (2)	0.001
*Cutibacterium* spp.	2 (2)	0 (0)	2 (3)	< 0.001
*Corynebacterium* spp.	1 (1)	1 (3)	0 (0)	0.323
Polymicrobial infection^c^	13 (14)	4 (13)	9 (14)	1.000
Negative culture infection	21 (23)	5 (17)	16 (25)	0.432
Site of pathogen isolation				
Blood culture	6/11 (55)	3/5 (60)	3/6 (50)	0.867
Intraoperative tissue culture	63/77 (82)	25/30 (83)	38/47 (81)	0.912

Data are *n*. (%) of episodes. The percentages were rounded and may not sum 100%. Whenever a denominator is shown, data are not available for all patients

^a^Among 32 *S. aureus* isolates, 4 (13%) were resistant to methicillin

^b^Including *S. epidermidis* (*n* = 13), *S. capitis* (*n* = 2) and *S. haemolyticus* (*n* = 1)

^c^Polymicrobial infections include *coagulase-negative staphylococci* (*n* = 6), *C. acnes* (*n* = 1), *Enterococcus* spp. (*n* = 6), *S. aureus* (*n* = 1), *Candida albicans* (*n* = 1), *Corynebacterium amycolatum* (*n* = 2), *Actinomyces* spp. (*n* = 1), *Finegoldia magna* (*n* = 1), *Enterobacter* spp. (*n* = 6), *E. coli* (*n* = 5), *Klebsiella* spp. (*n* = 1), *Peptostreptococcus* spp. (*n* = 1), *Streptococcus intermedius* (*n* = 1), *S. mitis* (*n* = 2), *Prevotella bivia* (*n* = 1), *Pseudomonas aeruginosa* (*n* = 1)

Blood cultures yielded the causative pathogen in 6 of 11 patients (55%), in whom blood cultures were collected, and intraoperative tissue cultures in 63 of 77 patients (82%), in whom these were collected. During the study period, no changes in the resistance against biofilm-active or other antibiotics were observed, e.g. rifampin-resistance in staphylococci remained < 3% and ciprofloxacin-resistance in gram-negative bacilli < 10% of clinical isolates.

### Surgical treatment

Table 4 shows the surgical treatment of patients with spinal implant-associated infections. All patients underwent at least one surgical intervention for treatment of spinal implant-associated infection. In 80 patients (86%) the implant was retained and a surgical debridement involving the spinal implant was performed. Retention of the implant was similarly distributed among patients treated with and without biofilm-active antibiotics (80% vs. 89%, respectively). The implant was partially or completely exchanged in one-stage procedure in 6 (6%). It was completely removed, without implantation of a new implant due to fused spine and bony stability in 7 patients (8%).

**Table 4:** Surgical treatment in 93 patients with spinal implant-associated infections

Surgical treatment	All patients (*n* = 93)	Patients treated with biofilm-active antibiotics (*n* = 30)	Patients treated with biofilm-non-active antibiotics (*n* = 63)	*p* value
Debridement and retention of implant^a^	80 (86)	24 (80)	56 (89)	0.338
One stage exchange of implant (partial or complete)	6 (6)	3 (10)	3 (5)	0.383
Complete removal of implant	7 (8)	3 (10)	4 (6)	0.677

Data are *n*. (%) of patients, unless otherwise indicated

^a^Among patients with retained implant, *S. aureus* was isolated in 8 of 24 patients (33%) with biofilm-active antibiotics and in 22 of 56 patients (39%) without biofilm-active antibiotics

### Evaluation of treatment outcome

After a median follow-up period of 53.7 months (range 8 days–9.4 years), 63 of 77 patients (82%) were infection-free, for whom follow-up data were available. One patient died 8 days after surgery because of non-infectious reason (cardiogenic shock), for 15 patients no follow-up data are available. There was no difference in the infection type and treatment modality in 15 patients lost to follow-up compared to the 77 patients with available follow-up.

**Fig. 1:**
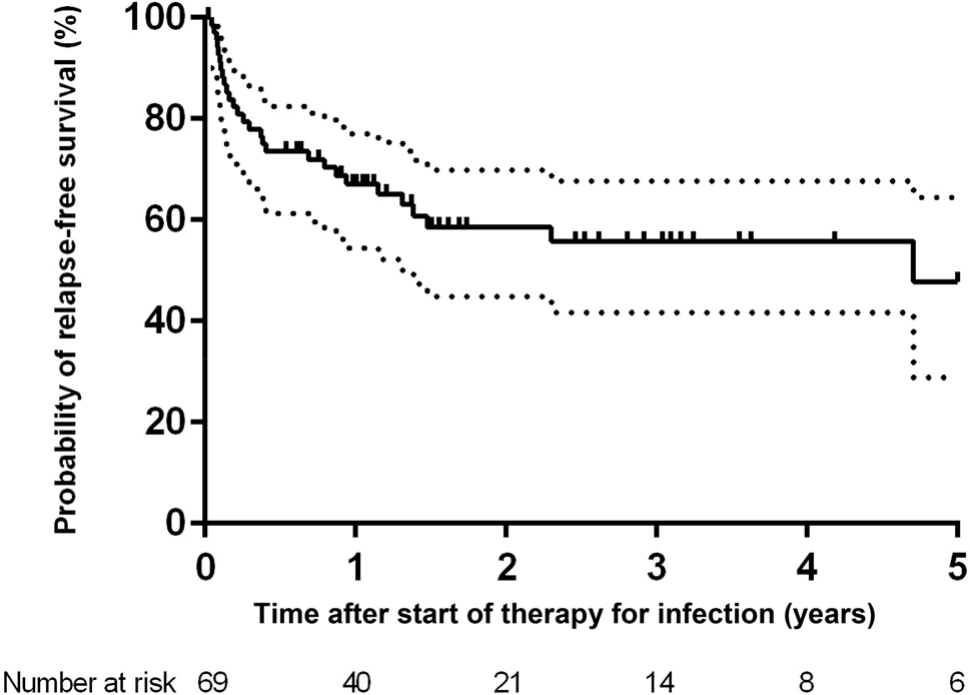
Kaplan–Meier survival curve of the estimated probability infection-free survival in 69 patients with implant-associated spinal infection. The vertical marks indicate censored events. The dotted lines represent 95% confidence interval

Figure 1 shows the estimated overall probability of infection-free survival, which was 67% (95% CI 55–82%) after 1 year and 58% (95% CI 43–71%) after 2 years. The median time from treatment of infection (first surgical intervention for infection) until relapse of infection was 105 days (range 16–1718 days).

**Fig. 2:**
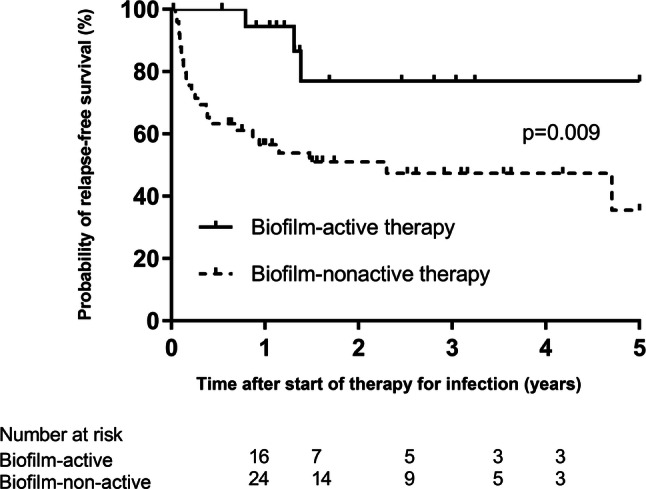
Kaplan–Meier curve of the estimated probability of infection-free survival time in 69 patients with implant-associated spinal infection, stratified for patients receiving biofilm-active and biofilm-nonactive antimicrobial therapy. The vertical marks indicate censored events

Figure 2 shows the estimated probability of infection-free survival for patients receiving biofilm-active and biofilm-nonactive antimicrobial therapy. The infection-free survival for patients who received biofilm-active antibiotics was 94% (95% CI 85–99%) after 1 year and 84% (95% CI 71–93%) after 2 years, whereas it was 57% (95% CI 39–80%) after 1 year and 49% (95% CI 28–61%) after 2 years for those who received no biofilm-active antibiotics.

In univariate outcome analysis (Table 5), biofilm-active antimicrobial treatment was associated with better outcome (HR, 0.23; *p* = 0.017), whereas isolation of *S. aureus* (HR 2.19, *p* = 0.039) and polymicrobial infection (HR, 2.44; *p* = 0.045) were associated with worse outcome. In multivariate analysis, only biofilm-active antibiotic therapy (HR, 0.24; 95% CI 0.07–0.79; *p* = 0.019) remained significantly associated with treatment outcome.

**Table 5:** Univariate analysis of factors associated with treatment failure in patients with spinal implant-associated infections

Factors	Hazard ratio (95% CI)	*p* value
Patient-related factors		
Age (per 1-year-increase)	1.01 (0.98–1.03)	0.678
Female gender	0.83 (0.40–1.75)	0.628
Number of comorbidities (per 1 increase)	1.17 (0.90–1.52)	0.251
Infection-related factors		
Treatment with vs. without biofilm-active antibiotic^a^	0.23 (0.07–0.77)	0.017
Early-onset vs. late-onset infection	0.66 (0.31–1.40)	0.278
Serum CRP value at discharge > 10 mg/l	1.30 (0.39–4.36)	0.666
Microbiology-related factors		
*Enterococcus* spp. vs. other pathogens	0.24 (0.03–1.81)	0.166
*S. aureus* vs. other pathogens	2.19 (1.04–4.62)	0.039
Polymicrobial versus monomicrobial infection	2.44 (1.09–6.04)	0.045
Coagulase-negative staphylococci vs. other pathogens	0.64 (0.22–1.85)	0.410
Surgical-related factors		
Number of segments stabilized (per 1 increase)	0.93 (0.79–1.10)	0.406
Indication for primary spinal surgery		
Degenerative spinal disease	0.72 (0.34–1.54)	0.393
Vertebral fracture	1.15 (0.45–2.94)	0.763
Spinal tumor	1.92 (0.77–4.76)	0.160
Vertebral osteomyelitis	0.61 (0.14–2.59)	0.502
Congenital deformity	1.15 (0.27–4.86)	0.850
Level of spine stabilization		
Cervical	2.39 (0.71–8.00)	0.159
Thoracic	0.72 (0.21–2.41)	0.589
Lumbosacral	1.31 (0.61–2.81)	0.489

*CRP* C-reactive protein, *95% CI* 95% confidence interval

^a^Biofilm-active antibiotics include a 12-week course of rifampin-combination for staphylococci, *Cutibacterium* spp. and culture-negative infections; or ciprofloxacin for gram-negative bacilli. For streptococci and enterococci, amoxicillin (or alternative active antibiotic) was used for prolonged treatment course of 6–12 months. Difficult-to-treat infections were excluded from the study

**Fig. 3:**
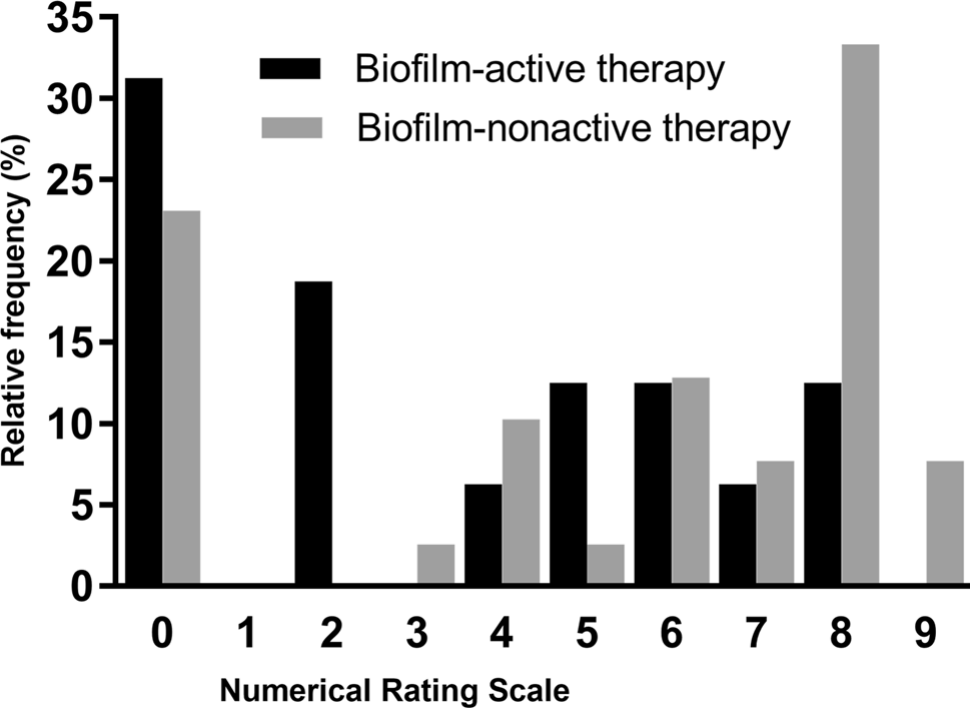
Numerical Rating Scale (NRS) evaluation of the pain at follow-up, stratified for patients receiving biofilm-active and biofilm-nonactive antimicrobial therapy

Figure 3 shows the pain score at follow-up among 56 patients. Patients with biofilm-active antimicrobial therapy reported lower intensity of postoperative pain. Severe pain (NRS > 7 points) was reported in 3 patients (18%) with biofilm-active antibiotic therapy and in 19 patients (49%) without biofilm-active antibiotic therapy (*p* = 0.027). 20 patients (51%) with biofilm-nonactive antimicrobial therapy and 14 patients (82%) with biofilm-active therapy were pain-free or had only mild or moderate pain (NRS ≤ 7 points). The mean number of subsequent surgical interventions was similar in patients treated with and without biofilm-active antibiotics (1.0 versus 3.4 interventions; *p* = 0.163).

Among 69 patients with follow-up, in 45 (65%) the implant remained in place. In patients with biofilm-active antimicrobial therapy, the implant was retained in 14 of 15 patients (93%) compared to 31 of 54 patients (57%) with a biofilm-nonactive antimicrobial treatment (*p* = 0.013). In 44 of 46 (96%) early-onset infections the implant was retained; of whom 38 (86%) were infection-free at last follow-up and retained the original implant in place. In contrast, in patients with late-onset infections, 15 of 23 patients (65%) were treated with implant retention, of whom 8 (53%) retained their implant in place at follow-up.

## Discussion

In this cohort study with 93 patients diagnosed with spinal implant-associated infections, most patients reported neck or back pain (75%) or local inflammatory signs (74%), whereas radiological signs of inflammation were present in only 40% of patients, in whom spine CT or MRI was performed. This finding supports the observation of Collins et al. [7], that isolated back pain was often the only consisting symptom of implant-associated infection.

The pathogen was mainly cultured from intraoperative tissue samples, demonstrating the importance of intraoperative sampling, as reported by other investigators [1, 5, 7, 16]. Due to the presence of spinal implants, low-virulent pathogens, such as coagulase-negative staphylococci and *Cutibacterium* spp. need to be considered, although they need to be distinguished from contaminants [27].

The overall infection-free survival rate of spinal implant-associated infections in our cohort was 67% after 1 year and 58% after 2 years. This survival rates are lower than in a previous study reporting infection-free survival rate of 85% after 1 year and 73% after 2 years [17]. In our study the treatment failure was defined more broadly, namely as implant removal for any reason or infection-related death. In other studies, some low-grade infections may have been missed and interpreted as mechanical implant failure. In another study [14], similar definitions for treatment failure were used as in ours and the infection-free survival was comparable (i.e. 66% after 2 years of follow-up).

The follow-up data were available for 77 patients (83%). Most infection relapses occurred within the first 2 years, therefore, the median follow-up period of 53.7 months (4.5 years) in this study seems appropriate to capture most infection relapses, except for low-grade infections, which may manifest several years later with presumed mechanical complications. Longer follow-up period is needed to answer this question.

In our cohort, biofilm-active antibiotic therapy was associated with better outcome and less pain severity than in patients without biofilm-active antibiotics. The implant was retained in most patients (86%), particularly in early-onset spinal infections. Multivariate analysis showed better outcome in patients treated with biofilm-active antibiotics (HR, 0.24). However, due to small number of patients it remains unclear, which patients may require longer or shorter duration of biofilm-active antibiotic treatment and whether differences between pathogens exist. The role of rifampin in the treatment of staphylococcal periprosthetic joint infections was previously demonstrated in several clinical studies [33].

While a strength of the study is the application of uniform definition criteria for diagnosis of infection and follow-up evaluation, the drawback is the non-randomized study design, heterogeneous surgical treatment modalities and high frequency of culture-negative infections. Furthermore, the low number of infections caused by streptococci (3 among polymicrobial infections) and enterococci (8 among monomicrobial and 6 among polymicrobial infections) makes any conclusions about the treatment outcome of these pathogens difficult, in particular because of prolonged antibiotic treatment of 6–12 months in these infections. Therefore, no conclusions can be made whether these infections are indeed eradicated or only suppressed, neither whether shorter or longer antibiotic therapy is required for infection-free status. Future studies should specifically address the pathogen-specific treatment in streptococci and enterococci, as well as their outcome. Another limitation of the study is that we excluded difficult-to-treat infections, for which no biofilm-active antibiotics exist. Despite in our study population the frequency of these pathogens is low, it may be higher in other regions. From this study no recommendation can be made regarding antibiotic treatment for these infections, neither whether suppression would work. Finally, the fact that a lower proportion of patients with degenerative spinal disease was included in the biofilm-active group and the lower pain intensity at follow-up may represent only a surrogate marker of lower prevalence of chronic pain disorder in this treatment group.

In conclusion, this study suggests that biofilm-active antibiotic therapy is associated with better treatment outcome and less postoperative pain compared to a treatment without biofilm-active antibiotics. These findings need to be confirmed in a randomized prospective study with larger patient numbers and longer follow-up period, in particular to evaluate potential low-grade infections.

## Electronic supplementary material

Below is the link to the electronic supplementary material.

Supplementary file1 (PDF 288 kb)
