# Supplementary material for: Outcome of spinal implant-associated infections treated with or without biofilm-active antibiotics: results from a 10-year cohort study
Source: Infection. 2020 May 5;48(4):559–68. doi: 10.1007/s15010-020-01435-2 (PMC7395063; doi:10.1007/s15010-020-01435-2)
Supplement: Supplementary file 1 — Supplementary file1 (PDF 288 kb) [file 15010_2020_1435_MOESM1_ESM.pdf]

Pocket Guide to Diagnosis & Treatment of Spinal Infections

PRO-IMPLANT FOUNDATION

Version 3: March 2020

For individual consultations and workshops contact our Consultation Portal at: cp.pro-implant.org

DEFINITION

Vertebral osteomyelitis is confirmed, if the following 3 criteria are fulfilled:

Investigation	Criteria
Clinical features	Acute or chronic back pain
Imaging	Computed tomography (CT) or magnetic resonance imaging (MRI) consistent with vertebral osteomyelitis
Microbiology<br>or<br>Histology	Microbial growth in blood culture or vertebral tissue[1]<br><br>Acute or chronic inflammation in vertebral tissue

Implant-associated spinal infection is confirmed, if ≥1 criterion is fulfilled:

Investigation	Criteria
Clinical features	• Wound healing disturbance or sinus tract (fistula)<br>• Visible purulence around the implant<br>• Positive „probe to implant“ test
Histology	Inflammation in peri-implant tissue
Microbiology	Significant microbial[2] growth in:<br>• ≥2 peri-implant tissue samples<br>• Sonication fluid (≥ 50 CFU/ml)

[1] Low virulent skin pathogens must be interpreted in the clinical context (prior infiltrations? Intravascular device present?)
[2] For highly virulent organisms (e.g. *S. aureus, E. coli*, streptococci) or patients under antibiotics already one positive sample confirms infection and <50 CFU/ml in sonication can be significant

Supportive criteria for infection:	
• Prolonged discharge from wound	• Loosening of the implant/screws
• Secondary wound dehiscence	• Non-union (pseudarthrosis)

Copyright: PRO-IMPLANT Foundation (N. Renz, S. Feihl, P. Vajkoczy, A. Trampuz). The Pocket Guide follows international recommendations. The Foundation cannot be held accountable for any treatment failures or antibiotic side effects. The latest version of the Pocket Guide is available at: www.pro-implant.org.
Contact email: info@pro-implant.org.

PATHOGENESIS

Post-interventional	Hematogenous	Contiguous
After<br>• Surgery/instrumen-tation<br>• Spinal infiltration/ injections<br>• Open/CT-guided biopsy	• Primary bacteremia/sepsis<br>• Urosepsis<br>• Endocarditis/infected intravascular device<br>• Dental focus (abscess/after interventions)<br>• Pulmonary infection<br>• Abdominal infection/tumor	• Skin infection<br>• Pressure ulcer<br>• Gastrointestinal sinus tract (oesophagus, intestine)<br>• Aortic infection

CLASSIFICATION

	Acute spinal infection	Chronic spinal infection
Pathogenesis		
▪ Post-interventional	<6 weeks after intervention (early)	≥6 weeks after intervention (delayed/low-grade)
▪ Hematogenous <u>or</u> contiguous	<6 weeks of symptom duration	≥6 weeks of symptom duration
Clinical features	Acute pain, fever, prolonged wound discharge (>7-10 days), acute neurologic impairment	Chronic pain, loosening/dislocation of the implant, sinus tract (fistula), neurologic impairment
Causative microorganism	High-virulent: *Staphylococcus aureus*, *Streptococcus* spp., gram-negative bacteria	Low-virulent: Coagulase-negative staphylococci, *Cutibacterium* (formerly *Propionibacterium*) *acnes*

MANAGEMENT ALGORITHM

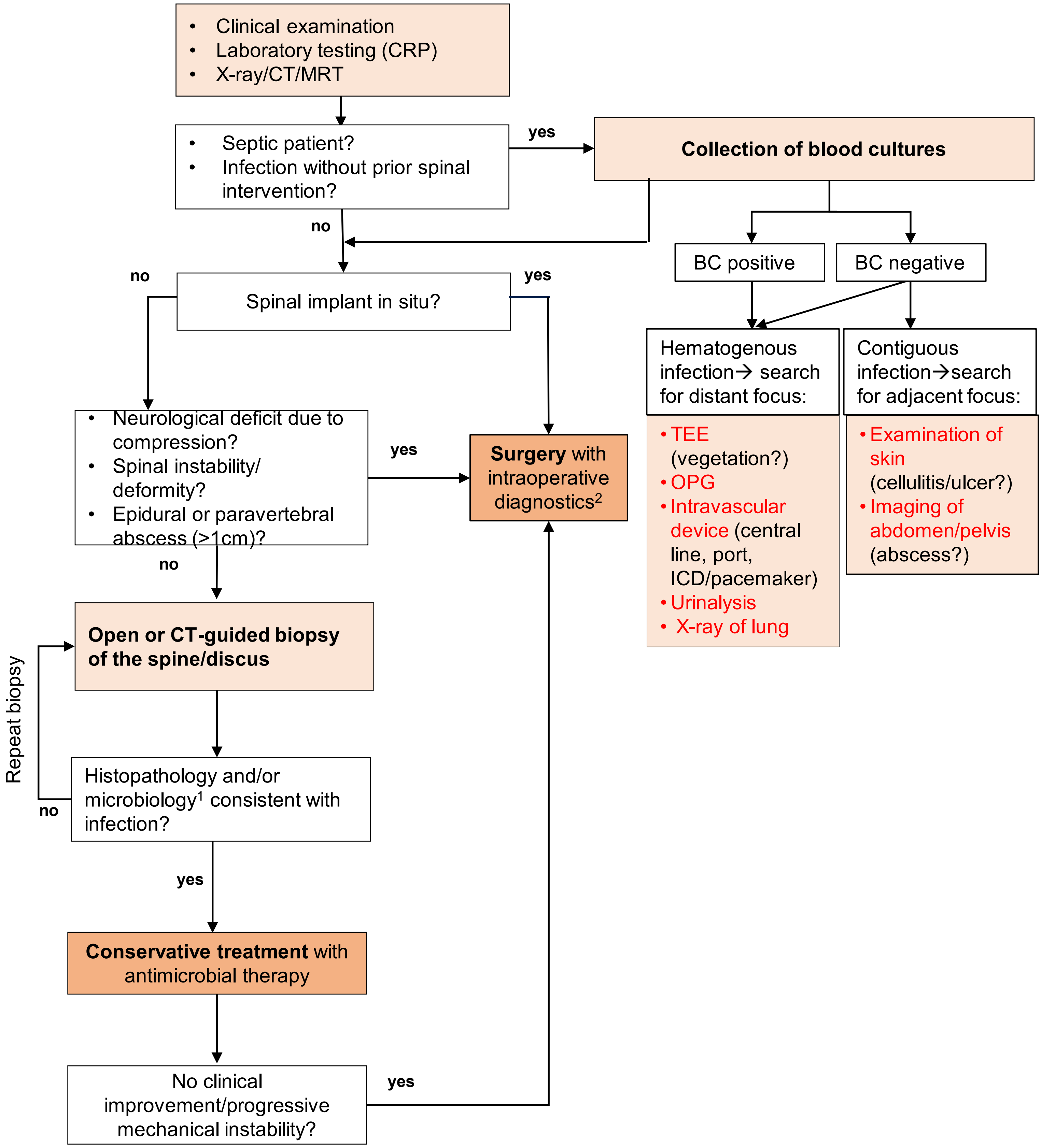

[1] For highly virulent organisms (e.g. *S. aureus, E. coli, streptococcus spp.)* or for patients on antibiotic therapy one positive sample confirms infection, for low-virulent organisms (e.g. *S. epidermidis, C. acnes*) ≥2 positive samples are required to confirm infection.

[2] See procedures on the following page. Histopathology, microbiology (+/- sonication), additional microbiological investigations (Mycobacteria, Brucella), if exposure/risk factors present.

TEE: transesophageal echocardiography, OPG: orthopantomogram, BC: blood cultures

SURGICAL PROCEDURES FOR IMPLANT ASSOCIATED SPINAL INFECTIONS

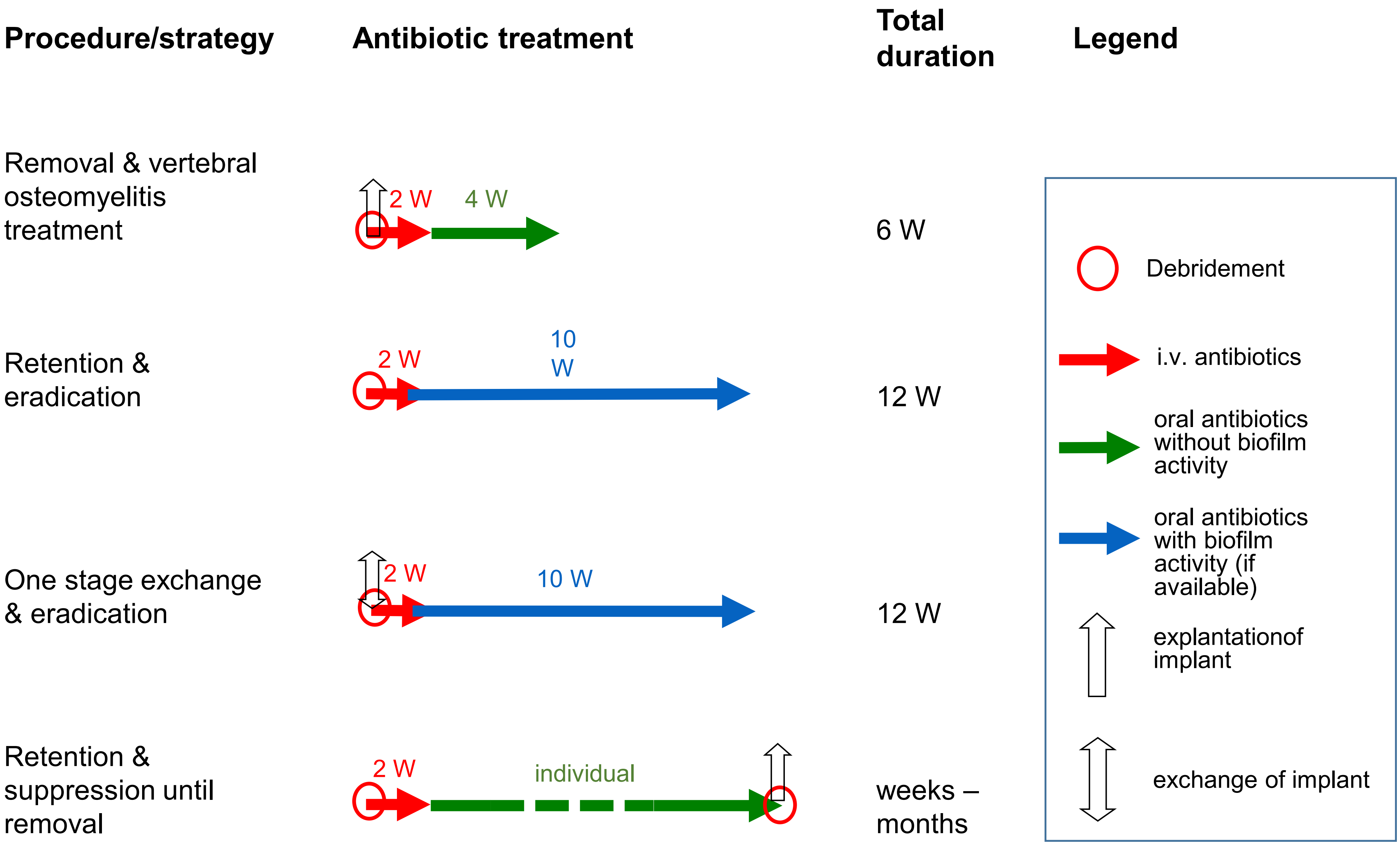

TREATMENT ALGORITHM FOR IMPLANT-ASSOCIATED SPINAL INFECTIONS

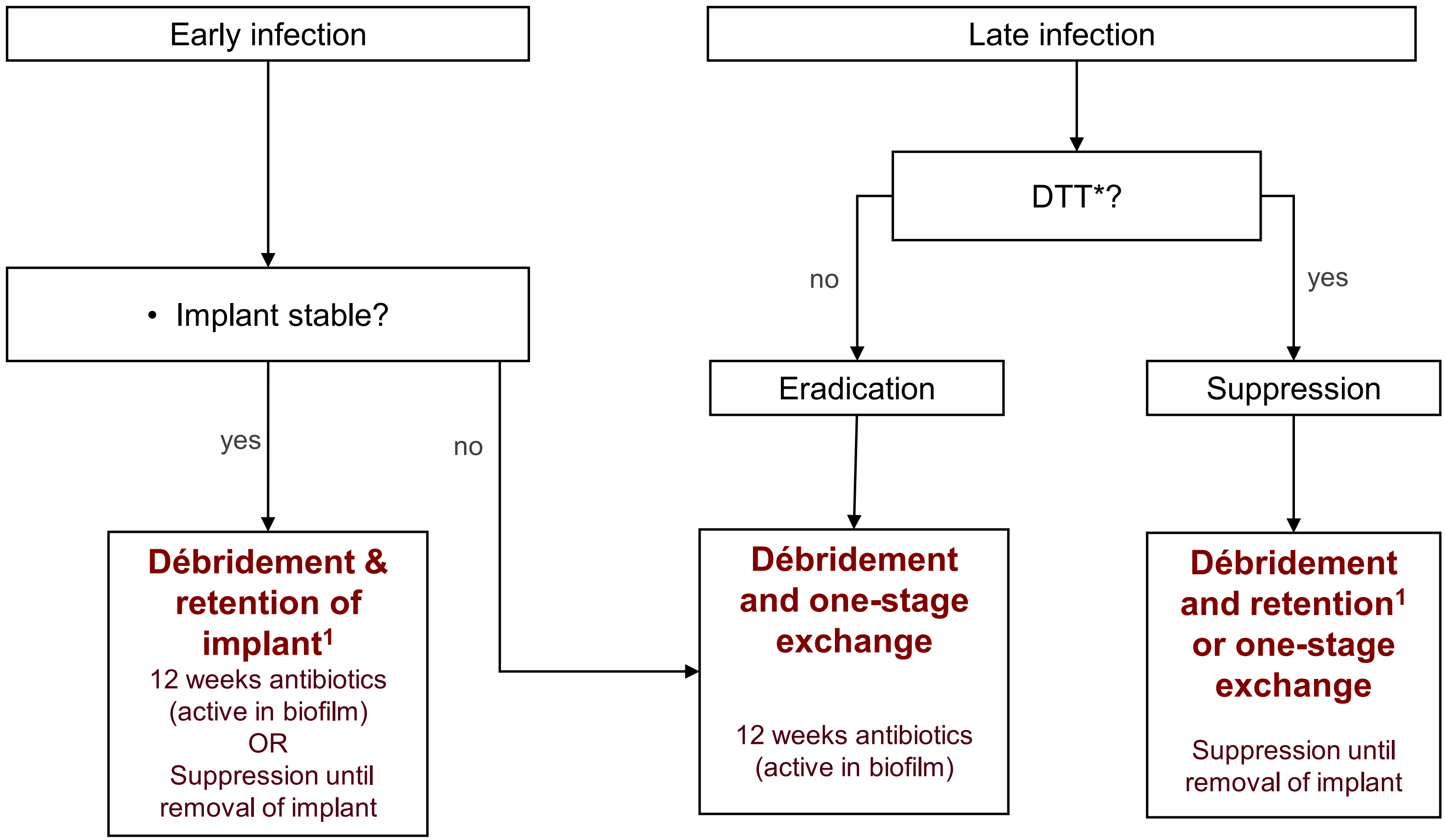

* DTT = difficult-to-treat, caused by pathogens resistant to biofilm-active antimicrobials:
- Rifampin-resistant staphylococci
- Ciprofloxacin-resistant gram-negative bacteria
- Fungi (Candida)

[1] exchange all loose parts (screws) if possible

RECOMMENDED ANTIMICROBIAL TREATMENT

Empiric therapy (intravenous):

Clinical situation	Primari focus	1st choice	Alternative
**Hematogenous vertebral osteomyelitis (without implant)**	Without known primary focus	Ampicillin/Sulbactam[a] 3 x 3 g	+ Fosfomycin 3 x 5 g (severe infection)
	Infectious endocarditis suspected	Ampicillin/Sulbactam[a] 4 x 3 g<br>+ Gentamicin[e] 1 x 240 mg	Ampicillin/Sulbactam[a] 4 x 3 g<br>+ Fosfomycin 3 x 5 g
	Primary focus in urogenital tract or abdomen suspected	Piperacillin/Tazobactam 3 x 4.5 g	Meropenem 3 x 1 g
	Allergy to penicillins:<br>- non-Type 1	Cefuroxim 3 x 1.5 g	Meropenem 3 x 1 g
	- Type 1 (anaphylaxis)	Vancomycin[d] 2 x 1g<br>+ Fosfomycin 3 x 5g	Daptomycin 1 x 8 mg/kg+<br>+ Fosfomycin 3 x 5g
**Post-interventional spinal infection (+/- implant)**	First revision	Ampicillin/Sulbactam[a] 3 x 3g<br>+ Fosfomycin 3 x 5g	Cefuroxim 3 x 1.5 g<br>+ Vancomycin[d] 2x1g
	Multiple previous revisions	Piperacillin/Tazobactam 3 x 4.5g<br>+ Fosfomycin 3 x 5g	Vancomycin[d] 2 x 1g<br>+ Fosfomycin 3 x 5 g

Targeted eradication therapy (deescalate as soon as the pathogen is known)**:**

**Microorganism** (red: difficult-to-treat)	**Antibiotic** (check pathogen susceptibility before)	**Dose**[b] (blue: renal adjustment needed)	**Route**
***Staphylococcus* spp.**			
- Oxacillin-/methicillin-susceptible	Flucloxacillin[a]	4 x 2 g	i.v.
	(+/- Fosfomycin)	(3 x 5 g)	i.v.
	for 2 weeks, followed by (according to susceptibility)		
	Rifampin[c] +	2 x 450 mg	p.o.
	- Levofloxacin or	2 x 500 mg	p.o.
	- Cotrimoxazole or	3 x 960 mg	p.o.
	- Doxycycline or	2 x 100 mg	p.o.
	- Fusidic acid	3 x 500 mg	p.o.
- Oxacillin-/methicillin-resistant	Daptomycin or	1 x 8 mg/kg	i.v.
	Vancomycin[d]	2 x 1 g	i.v.
	(+/- Fosfomycin)	(3 x 5 g)	i.v.
	for 2 weeks, followed by an oral **rifampin** combination as above		
- Rifampin-resistant	Intravenous treatment according susceptibility for 2 weeks (as above), followed by long-term suppression for ≥1 year (e.g. Doxycycline)		
***Streptococcus* spp.**	Penicillin G[a] or	4 x 5 million U	i.v.
	Ceftriaxone	1 x 2 g	i.v.
	for 2-3 weeks, followed by:		
	Amoxicillin or	3 x 1000 mg	p.o.
	Doxycycline (suppression ≥1 y.)	2 x 100 mg	p.o.
***Enterococcus* spp.**			
- Penicillin-susceptible	Ampicillin +	4 x 2 g	i.v.
	Gentamicin[e]	1 x 120 mg	i.v.
	(+/- Fosfomycin)	(3 x 5 g)	i.v.
	for 2-3 weeks, followed by:		
	Amoxicillin	3 x 1000 mg	p.o.
- Penicillin-resistant or allergy to penicillin	Vancomycin[d] or	2 x 1 g	i.v.
	Daptomycin	1 x 10 mg/kg	i.v.
	+ Gentamicin[e]	1 x 120 mg	i.v.

	(+/- Fosfomycin)<br>for 2-4 weeks, followed by<br>Linezolid (max. 4 weeks)	(3 x 5 g)<br><br>2 x 600 mg	i.v.<br><br>p.o.
- Vancomycin-resistant (VRE)	Individual; removal of the implant or life-long suppression necessary (e.g. with Doxycycline)		
**Gram-negative**			
- Enterobacteriaceae (*E. coli, Klebsiella, Enterobacter* etc.)	Ciprofloxacin[f]	2 x 750 mg	p.o.
- Nonfermenters (*Pseudomonas aeruginosa, Acinetobacter* spp.)	Piperacillin/tazobactam or<br>Meropenem or<br>Ceftazidim/Cefepime<br>+Tobramycin<br>(or gentamicin)<br>for 2-3 weeks, followed by:<br>Ciprofloxacin	3 x 4.5 g<br>3 x 1 g<br>3 x 2 g<br>1 x 300 mg<br>1 x 240 mg<br><br>2 x 750 mg	i.v.<br>i.v.<br>i.v.<br>i.v.<br>i.v.<br><br>p.o
- multiresistant	Depending on susceptibility: a combination of meropenem 3 x 1 g i.v., colistin 3 x 3 million U i.v. and/or fosfomycin 3 x 5 g i.v., consider oral suppression (if Cipro-R)		
**Anaerobes**			
- Gram-positive (*Cutibacterium, Peptostreptococcus, Finegoldia magna*)	Penicillin G[a] or<br>Ceftriaxon<br>for 2 weeks, followed by:<br>Rifampin[c] +<br>Levofloxacin or<br>Amoxicillin	4 x 5 million U<br>1 x 2 g<br><br>2 x 450 mg<br>2 x 500 mg<br>3 x 1000 mg	i.v.<br>i.v.<br><br>p.o.<br>p.o.<br>p.o.
- Gram-negative (*Bacteroides*)	Ampicillin/sulbactam[a]<br>for 2 weeks, followed by<br>Metronidazol	3 x 3 g<br><br>3 x 400 mg or 500 mg	i.v.<br><br>p.o.
Candida spp.<br>- Fluconazole-susceptible	Caspofungin[g] or<br>Anidulafungin<br>for 2 weeks, followed by:<br>Fluconazole<br>(suppression for ≥1 year)	1 x 70 mg<br>1 x 100mg (1. Day 200mg)<br>1 x 400 mg	i.v.<br>i.v.<br>p.o.
- Fluconazole-resistant	Individual (e.g. with voriconazole 2 x 200 mg p.o.); removal of the implant or long-term suppression		
**Culture-negative**	Ampicillin/sulbactam[a]<br>for 2 weeks, followed by:<br>Rifampin[c] +<br>Levofloxacin	3 x 3 g<br><br>2 x 450 mg<br>2 x 500 mg	i.v.<br><br>p.o.<br>p.o.

[a] **Penicillin allergy** NON-type 1 (e.g. skin rash): cefazolin (3 x 2 g i.v.). In case of anaphylaxis (= type 1-allergy such as Quincke´s edema, anaphylactic shock) or cephalosporin allergy: vancomycin (2 x 1 g i.v.) or daptomycin (1 x 8 mg/kg i.v.) Ampicillin/sulbactam is equivalent to amoxicillin/clavulanic acid (3 x 1.2 g or 3 x 2.2 g i.v.)

[b] **Laboratory testing** 2x weekly: leukocytes, CRP, creatinine/eGFR, liver enzymes (AST and ALT). Dose-adjustment according to renal function and body weight (<40 or >100kg)

[c] **Rifampin** is administered only when a newimplant is in situ. Add it orally to i.v.-treatment as soon as wounds are dry; in patients aged >75 years, rifampin is reduced (2 x 300 mg).

[d] Check **Vancomycin** concentration at least 1x/week(take blood before next dose); therapeutic range: 15-20 µg/ml

[e] Give gentamicin only, if **gentamicin high-level (HL)** is tested susceptible (consult your microbiology laboratory). In gentamicin HL-resistant *E. faecalis* or patients with impaired renal function: gentamicin is exchanged with ceftriaxone 1 x 2 g i.v. or Fosfomycin 3 x 5 g i.v.

[f] Add **i.v. treatment** (piperacillin/tazobactam 3 x 4.5 g or ceftriaxon 1x 2 g or meropenem 3 x 1 g i.) in the first postoperative days (until wound is dry)

[g] In patients weighing <80 kg: a loading dose of 70 mg on day 1, then **reduce to 50 mg** from day 2.

Oral therapy in vertebral osteomyelitis WITHOUT implant

**Microorganism**	**Antibiotic** (according to susceptibility, for dose see table above)
S. aureus	Rifampin combination (see above)
Coagulase-negative staphylococci	Cotrimoxazole or doxycycline or clindamycin
Streptococcus spp.[1]	Amoxicillin or clindamycin or doxycycline
Enterococcus spp.[1]	Amoxicillin (or linezolid, if resistant to amoxicillin)
Anaerobes (gram-positive)	Clindamycin or amoxicillin
Anaerobes (gram-negative)	Metronidazole or clindamycin
Gram-negative bacilli	Ciprofloxacin or cotrimoxazole
Fungi (*Candida* spp.)	Fluconazole (voriconazole if fluconazole resistant)

[1] Consider longer intravenous treatment (4 weeks) in *S. agalactiae, S. dysgalactiae* and enterococci.

Treatment duration:

- Native spinal infection (WITHOUT Implant): 6 weeks (in most cases but longer, if non-drained abscess)
- Implant associated spinal infection: 12 weeks (in most cases)

Follow-up:

- clinical and laboratory (CRP) course
- NO scheduled CT or MRT imaging, only if new symptoms occur or clinical course is unfavorable

INVESTIGATION OF PRIMARY FOCUS

**Pathogen**		**Primary focus**	**Investigation**
**Staphylo-coccus spp.**	*S. aureus*	• Skin lesions/furunculosis<br>• Endocarditis<br>• Primary bacteremia	• Skin examination<br>• Blood cultures<br>• Transesophageal echocardiography (TEE)
	Coagulase-negative staphylococci	• Intravascular implant<br>• Endocarditis	• Blood cultures, TEE<br>• Intravascular implant in situ?
**Strepto-coccus spp.**	Viridans group (*S. mitis/oralis*)	• Oral cavity<br>• Endocarditis	• Orthopantomogram<br>• Blood cultures, TEE<br>• Recent dental procedure?
	S. agalactiae,<br>*S. dysgalactiae*	• Abdomen<br>• Urogenital tract<br>• Skin<br>• Oral cavitiy	• Imaging of abdomen/pelvis<br>• Urinalysis<br>• Skin examination<br>• Orthopantomogram
	S. gallolyticus (formerly *bovis*)	• Colon carcinoma /adenoma	• Colonoscopy
**Entero-coccus spp.**	*E. faecalis,*<br>*E. faecium*	• Abdomen<br>• Urogenital tract<br>• Endocarditis	• Imaging of abdomen/pelvis<br>• Urinalysis<br>• Blood cultures, TEE
**Gram-negative rods**	*E. coli, Klebsiella, Enterobacter* spp.	• Abdomen<br>• Urogenital tract	• Imaging of abdomen/pelvis<br>• Urinalysis<br>• (Colonoscopy)
